# The Sandow Case

**Published:** 1911-06-03

**Authors:** 


### The Hospital

A Journal of

The Medical Sciences and Hospital Administration.

New Series. No. 226, Vol. IX. [No. 1298, Vol. L.] SATURDAY, JUNE 3 1911

### The Sandow Case.

On Friday, May 26, the General Medical Council concluded its investigation of the charges brought by the British Medical Association against three medical men who have been associated with the Sandow "Curative Institute." The upshot of the proceedings was that Mr. Maurice Edmund Arnold Wallis was declared guilty of infamous conduct in a professional respect, and that the Registrar was directed to erase his name from the Medical Register. In the cases of the other respondents, Messrs. James Robertson Wallace and Charles Edward Trimble, the facts alleged in the complaint were proved to the satisfaction of the Council, but judgment was postponed until the next sessions in order that the defendants might reconsider their position.

Thus ends, for the time being at least, an anomaly which has for long been permitted to continue unchecked. Whether Mr. Sandow retains the services of these practitioners, whether he finds others equally careless of the penalties of erasure from the Register, or whether he dispenses with medical supervision altogether, at least it will not be possible for him to pretend that his "treatment" has the sanction of the Medical Council. During the hearing of the case, the counsel for the defence, Lord Robert Cecil, was very scornful of Mr. Sandow's exaggerated claims, and of the strong savour of quackery pervading the whole establishment. That, however, was with the view of showing that his clients really had no association with Mr. Sandow except that of employés paid to decide whether each individual patient was in a fit state to undertake a course of exercises or not. He emphasised the point that the defendants took no part in prescribing treatment and had no share in the management or profits of the concern. All this may have been good pleading on behalf of the three practitioners charged, but it makes the case against Mr. Sandow himself all the stronger and more urgent. As a matter of fact, the defence had to admit that many patients were dealt with by correspondence, and that their diagnoses of their own cases were accepted unless those of their regular medical attendants could be obtained. These admissions speak volumes for the contentions of the prosecution. One of our contemporaries has on former occasions courageously taken up the cudgels for the profession against Mr. Sandow. But it is time that the General Medical Council, the Medical Defence Union, or some similar organised and efficient medical society, should move. The gravamen of the matter is not that Mr. Sandow does no good; on the contrary, so long as a large section of the community eats too much and takes too little exercise, any "professor" of physical culture will do many of his clients good. That Mr. Sandow's methods of physical culture are good is not the point. The onus of the charge which he has to meet is that he has long ago overstepped the legitimate limits of a physical culturist, and that he professes and practises the cure of disease without any real knowledge of either disease itself or the effects upon it of exercises. It may be inferred from the arguments of the defence in the recently-concluded case, that he regards himself as the prime agent, or healer, of those who seek his treatment. He employs the three gentlemen who have been before the General Medical Council, more to satisfy the conventions and the law of the land than because he really wants their assistance. In other words, he is acting the part of an unqualified practitioner; and, since those advertisements, which Lord Robert Cecil admitted are flamboyant and ludicrous, confront the world in every magazine and on whole sheets of the daily papers, it is fair to add that he pretends to a knowledge of disease and cure exceeding that of the qualified medical practitioner. Whether he really deludes himself with this idea, or merely assumes the pose, does not affect the matter a jot: the fact remains that he is an unqualified practitioner whose operations are a menace to the interests of the community in so far as they include the treatment of disease by one whose knowledge of the subject is as limited as his advertisements are comprehensive. Let us repeat that with Mr. Sandow, the physical culturist, the profession has no quarrel: quite the reverse, indeed. But for Mr. Sandow, the quack doctor, "covered" by the connivance of members of the medical profession, no condemnation can be too strong. The basis of the case against him can easily be found in the speech of Lord R. Cecil, appearing, be it remembered, on behalf of Mr. Sandow's medical staff. In no ordinary sense, he explained, is the treatment such as requires medical skill to administer; and he went on to describe the advertisements as pernicious, blatant, and foolish. Finally, he offered, on behalf of Mr. Sandow, to refrain from further advertisement, a clear admission that these advertisements, with which the country has been deluged for years, form a spot in his armour so weak that in view of what has happened to the three medical men it is considered to be no longer advisable to offer it for attack. Perhaps with a little further pressure it might be possible to get Mr. Sandow to resume his proper sphere of expert in physical culture and to abandon that of unqualified medical practice altogether.

